# *Cannabis sativa* L. may reduce aggressive behaviour towards humans in shelter dogs

**DOI:** 10.1038/s41598-021-82439-2

**Published:** 2021-02-02

**Authors:** Sara Corsetti, Simona Borruso, Livia Malandrucco, Valentina Spallucci, Laura Maragliano, Raffaella Perino, Pietro D’Agostino, Eugenia Natoli

**Affiliations:** 1 School of Agriculture and Environment, The University of Western Australia, Crawley, WA 6009 Australia; 2 Canine Consultant, Rome, Italy; 3 Canile Sovrazonale, ASL Roma 3 (Local Health Unit Rome 3), Rome, Italy; 4 Istituto Zooprofilattico Sperimentale del Lazio e della Toscana M. Aleandri, Rome, Italy; 5 Canile Pubblico Muratella e Pontemarconi, Roma Capitale (Municipality of Rome), Rome, Italy

**Keywords:** Animal behaviour, Quality of life

## Abstract

Among the phytocomplex components of *Cannabis sativa* L*.*, cannabidiol (CBD) has a recognised therapeutic effect on chronic pain. Little is known about the veterinary use of CBD in dogs. Even less is known on the effects of CBD on dog behaviour, especially in shelters. The purpose of this study was to determine if CBD affects stress related behaviour in shelter dogs. The sample consisted of 24 dogs divided into two groups that were created by assigning the dogs alternately: 12 dogs were assigned to the treatment group and 12 to the control group. Extra virgin olive oil, titrated to 5% in CBD was given to treated group; the placebo consisted of olive oil only, dispensed daily for 45 days. Behavioural data were collected using the ‘focal animal’ sampling method with ‘all occurrences’ and ‘1/0’ methods for 3 h: before (T_0_), after 15 days (T_1_), after 45 days of treatment (T_2_) and after 15 days from the end of the treatment (T_3_). Treated dogs showed reduced aggressive behaviour toward humans following the treatment (Friedman Test: χ^2^ = 13.300; df = 3; N = 12; p = .004; adj. sig. p = 0.027), but the difference in the decrease of aggressive behaviour between the two groups was not significant (Mann–Whitney U test, T_2_–T_0_: Z = − 1.81; N = 24; p = 0.078). Other behaviours indicative of stress, such as displacing activities and stereotypes, did not decrease. Despite some non-significant results, our findings suggest that it is worth doing more research to further investigate the effect of CBD on dog behaviour; this would be certainly valuable because the potential for improving the welfare of dogs in shelters is priceless.

## Introduction

*Cannabis sativa* L*.*, also commonly known as hemp, has provided fabric, oils, food and rope for humans for thousands of years^1,2^. It has also been widely used for its medical and psychoactive effects^1,2^. It has more than 489 chemical compounds including terpenes, hydrocarbons, ketones, aldehydes and phytocannabinoids^3^. The two best known cannabinoids are cannabidiol (CBD) and tetrahydrocannabinol (THC). While the second one is responsible for the psychotropic and toxic effect, both in humans and animals (e.g.,^4–7^), CBD has no psychotropic effects and has a low toxicity^8–10^. Due to its high tolerability^8^, it has been increasingly used in clinical trials for humans and animals (e.g.,^11–13^).

Despite the discomfort that many veterinarians feel in proposing cannabis-derived remedies to pet owners^14^, CBD is gradually becoming an important tool for the treatment of pain, inflammation, seizures and anxiety (e.g.,^14–16^). In 2019,^14^ 1940 veterinaries were interviewed: of these, 1806 (93,1%) discussed the use of CBD with owners for management of pain, 1341 (69,1%) for anxiety and 1089 (56,1%) for seizures. Although the use of cannabinoid products to treat animals’ behavioural problems in domestic animals has been recently increasing^17,18^, there is scarce literature on clinical trials to evaluate its effectiveness. Deiana et al*.*^19^ tested different compounds of *Cannabis sativa*, finding that CBD reduced obsessive-compulsory behaviour in rats and mice. In the same year, another study showed that administration of CBD reduced marble-burying behaviour in mice^20^.

Few studies have assessed the effect of CBD on dog health and behaviour. Deabold et al*.*^13^ studied the pharmacokinetics of CBD in dogs and cats. Their results suggest that orally administered CBD in dogs was not detrimental with a time gap of 12 h or more between one administration and another. Similar results were found by McGrafth et al.^16^: dogs tolerate CBD well if fasting and postprandial bile acids remained stable. Gamble and collaborators^15^ found that a CBD-based treatment decreased pain and increased activity in dogs with osteoarthritis.

CBD interacts with organisms through the endocannabinoid system (ECS). In vertebrates and invertebrates, the animal’s ECS is a biological system interacting with both endogenous cannabinoids and the exogenous plant molecules derived primarily from hemp^21^. The ECS owes its name to the previous discovery of some elements’ ability, which constitute it, to interact with THC. In mammals, the ECS is very complex and modulates different kind of organism responses^21^. Through the two principal receptors (CB1 and CB2), it takes part in the anti-inflammatory process^22^, in the management of anxiety^23^, in the immune function^12,24^ and in lowering pain^25^. This system is also involved in maintaining homeostasis for different organs and in modulating the nervous and immune systems^21^. Even if^26^ and^27^ demonstrated that CBD has a low affinity for CB receptors, it is an agonist of 5-HT_1A_ receptors^28^. These receptors are part of a class of receptors (5-HT) that usually interact with serotonin^29^ and are strictly associated with physical health^30^, mood^30^ and stress [reviewed in^31^].

Stress is a mental, physiological, or emotional state characterized by a factor that is altering the homeostasis of a living organism^32^. For mammals, the response to a stressor, which can be physical or emotional, as for example infections, burns or anger^33^ involves the hypothalamic–pituitary–adrenal axis reactivity (e.g.,^34^), resulting in an increase of circulating glucocorticoids that could result in stress-related disorders^35^.

For dogs, entering a kennel represents a stressful event (e.g.,^36–43^) due to several stressors including exposure to a new context or social and spatial restrictions (e.g.,^44^). In many countries like Italy, where sheltered dogs cannot be euthanased except for health reasons or proven dangerousness according to the law, it is our duty to guarantee them an acceptable level of well-being. There is still a debate on behavioural indicators of dogs’ low level of welfare when in kennels^45^; however, there is no doubt that displacing activities^46–48^ and stereotyped behaviours^47–49^ are both indicators of moderate to high level of anxiety, and consequently discomfort, as well as of pathological behaviour in dogs; in addition, persistent aggressive behaviour, out of context, can be considered a pathological behaviour^50^. As defined in^46^, ”Displacing activities are behaviour patterns (mostly body care activities) characterized by their apparent irrelevance to the situation in which they appear. […] Displacement activities tend to occur in situations of psycho-social stress”. Aggressive behaviour is part of all species' behavioural repertoire; the ultimate causes that led to its evolutionary selection concern function in intra-and inter-specific competition^51^; in other words, aggressive behaviour has evolved to allow individuals to be competitive for obtaining the resources necessary for their survival^52^.

Some psychoactive medications, including herbal supplements or pheromonal products, have been used to lower the level of anxiety of dogs (e.g.,^53,54^), but no other studies have evaluated the influence of CBD on dog behaviour. The study was a clinical trial and its purpose was to determine if CBD treatment can decrease disturbed and stressed behaviour in shelter dogs, in terms of decrease in displacing activities, stereotyped and aggressive behaviour.

## Materials and methods

### Animals and housing

The subjects of this study were 24 domestic dogs (20 neutered males, 2 unneutered males, 2 spayed females) with various kind of behavioural problems, randomly drawn from a list of animals matching the inclusion criteria. The behavioural problems were diagnosed by the kennel’s veterinarians working for the Local Health Unit and the Municipality of Rome. The criteria for selection were: age between 1 and 10 years (estimated by standard veterinary methods); physically healthy; presence of behavioural disorders (detected by the veterinarian); permanence in the shelter for at least 9 months (Table 1). The latter item was included in the criteria to avoid biasing the results by measuring behavioural responses due to acute stress; in fact, the literature reports that dogs entering the shelter have different behavioural, physiological and immunological responses due to acute stress^36,45^. The different sex ratio of the selected dogs was due to the shortage of females that met the parameters for the selection and, at the same time, presented behavioural problems. Eighteen of the dogs were mixed-breed and six were clearly purebred-derived dogs (one Bull Terrier, one Bull Mastiff, one Italian Mastiff, three American Pit Bull Terrier).

**Table 1:** The 24 dogs selected for the study, their weight, principal behavioural disorder, group and dosage.

Dog name	Weight (kg)	Behavioural disorder	Group	Dosage (drops)
Sonny	28	Licking bars of the cage	Treatment	13
Willy	37	Coprophagy and fearful	Treatment	17
Nerone	27	Obsessive jumping and pacing in circles	Treatment	13
Caos	30	Pacing in circles	Treatment	15
Tacchino	27	Drooling	Treatment	13
Bullo	26	Fearful and aggressive	Treatment	12
Gargamello	17	Aggressive	Treatment	8
Creamy	30	Aggressive	Treatment	15
Gaemon	32	Fearful	Treatment	16
Teddy	16	Aggressive	Treatment	8
Gastone	32	Fearful	Treatment	16
Oreste	36	Aggressive	Treatment	17
Orco	41	Fearful	Placebo	20
Sid	19	Coprophagy and obsessive jumping	Placebo	10
Pongo	24	Aggressive	Placebo	12
Mina	37	Fearful	Placebo	17
Golia	33	Fearful	Placebo	16
Ulisse	42	Licking bars of the cage	Placebo	20
Cagnaccio	25	Aggressive and drooling	Placebo	12
Rocky I	22	Aggressive	Placebo	11
Camelio	30	Aggressive	Placebo	15
Macchia	32	Pacing in circles	Placebo	15
Rocky II	24	Aggressive	Placebo	12
Piso	21	Fearful	Placebo	12

The selected dogs showed severe behavioural disorders such as compulsively licking the cage walls, chewing on objects until they were destroyed, coprophagy or having attacks of aggression such as to lead to self-injury; none were under therapeutic, pharmacological or behavioural treatment.

Every day the shelter operators monitored the dogs to spot symptoms (vomiting, diarrhoea) of possible health issues; such occurrences were registered and reported to the responsible veterinarian.

The study was carried out in the dog shelter “Muratella”, the municipal dog shelter in Rome. The dogs were housed in single cages of 4 m^2^ with an indoor and outdoor area. The cages were cleaned twice a day, before food distribution. All the dogs could go out in a fenced area (10 × 3 m) adjacent to their cages. A few of them were taken out for a walk inside the shelter by the staff and/or volunteers. Given that changing dogs’ daily routines might be an additional source of stress for them^55^, we maintained their lifestyles through the study.

### Treatment

We calculated that the minimum sample size (shelter dogs’ population = 400; prevalence of stress signals in shelter dogs = 90%; power = 0.80; alpha error = 5%; n1/n2 = 1) was 10 individuals in each group, alternately assigned (group A = treated; group B = control); we include two additional individuals for each group to address possible drop outs.

The dogs belonging to the treatment group were given a CBD based oil while the dogs belonging to the control group were given a placebo. Both were administered every day before the usual meal in the morning, for 45 days. CBD based oil consisted of an extraction from aerial parts and inflorescences of the plant *Cannabis Sativa* in organic extra virgin olive oil to the proportion of 150 g of *Cannabis Sativa* inflorescences and aerial parts in 1 L of oil. The extraction was done using the “Naviglio” extractor, titrated to 5% in CBD and THC absence. The placebo consisted of extra virgin olive oil only.

The dosage to each dog was calculated as follow: 1 drop of oil/2 kg of weight, i.e. 5 drops of oil were administered to a dog that weighed 10 kg, 10 drops to a dog that weighed 20 kg and so on. The percentage of body fat was calculated for each dog by means of the conditional body score (BCS): in case of obesity, dogs were given an extra 20% of drops (Table 1).

With and without CBD, the oil administration did not require any kind of particular interaction since the oil was mixed with some meat; in any case, due to their behavioural disorders, most of the dogs did not allow any form of interaction with humans. However, the operators were instructed not to alter the usual quantity and quality of daily interactions.

### Behavioural observations

The observations were carried out live by two previously trained observers, blind to which group (treated or control) the dogs belonged to; an inter-observer reliability test was conducted prior to the trial. The behavioural observations were conducted by a single observer each time who sat in front of the cage; observers did not interact with the dogs, so the dogs became rapidly accustomed to the presence of the observers. The time period of observations ranged from September to December 2018. The 24 dogs were observed exclusively in their home-cage for 12 h each, for a total of 288 h. Before starting the administration of CBD based oil and the placebo, each dog was observed for one hour a day for three consecutive days (T_0_), at three different times of the day (morning, between 8:00 A.M. and 12:00 P.M. hours; lunchtime, between 12:00 P.M. and 3:00 P.M. hours; late afternoon, between 3:00 P.M. and 7:00 P.M. hours). Twenty-four hours after the last day of T_0_, the treatment began.

The collection of behavioural data was repeated in the same way in the following intervals: from the 15th to the 17th day (T_1_) and from the 43rd to the 45th day (T_2_) of the administration of treatment; from the 15th to the 17th day (T_3_) after the end of the treatment.

The ethogram utilised for data collection during behavioural observations consisted of more than 100 behavioural patterns (described previously in^43^, see Supplementary Information): by means of the focal animal sampling method^56^, the behavioural patterns of each dog were recorded in a check sheet, utilising the “all occurrences” and “1/0” methods (60 s interval)^56^. The “all occurrences” method provides the number of times a dog shows a specific behaviour (for example the number of times it scratches himself), while the 1/0 method gives the number of predetermined intervals (in this case 60 s) in which the dog exhibits a behaviour (e.g., the number of intervals in which the dog barks)^56^.

### Statistical analysis

The behavioural patterns utilised to collect data during the observations were grouped into categories (Table 2), generated on the basis of information drawn from the literature^41,42^ and repeatedly used in the past by our working group^43,57,58^. Since the numbers were not normally distributed, to compare the behavioural frequencies recorded in the different times (T_0_, T_1_, T_2_, T_3_) for the control and treatment groups separately, we utilised the Friedman test, a non-parametric alternative for a repeated-measures ANOVA, and the Bonferroni correction for multiple comparisons. To compare the difference between treated and placebo group, we utilised the Mann–Whitney U test. A p value of < 0.05 was used to determine significance.

**Table 2:** The behavioural patterns utilised in this study grouped into categories. For the description of the behaviours, see Supplementary Information.

Behavioural category	Observed behavioural patterns
Activity	Standing, walking, trotting, galloping, in/out from the internal to the external area of the cage and vice-versa
Aggressive behaviour	Growling, sideways glance, raising fur, curling lip, showing teeth, dashing at bars
Displacing activities	Body shaking, scratching, muzzle licking, auto-grooming
Stereotyped or repetitive behaviour	Repetitive pacing in circles, licking or biting compulsively, catching flies, coprophagy, obsessed with an object, self-mutilation
Attention	Raising ears, looking outside, looking out carefully, looking at observer, looking at unknown people, looking at volunteer, looking at dog, raising foreleg, raising forelegs on wall
Olfactory investigation	Sniffing environment, sniffing air, sniffing observer, sniffing unknown people, sniffing volunteer, sniffing dog
Dominant behaviour	Staring, stiff body and tail still, raised tail, wagging with the tail held high, pricked-up ears, paw or a muzzle on a conspecific’s back
Submissive behaviour	Avoiding eye contact, ears down, cringing, tail between the legs, lying down on the back
Vocal communication	Barking, whining, grumbling, mumbling, howling, snorting
Affiliative behaviour	Wagging tail, offering the front paw, leaning on bars
Resting	Sitting, lying, dozing
Playing	Inviting to play, answering invitation to play

Data analysis was conducted using the IBM SPSS software.

### Ethics statements

This study was approved by the Animal Welfare and Protection Office of the Municipality of Rome, which is responsible for sheltered dogs according to Italian laws, and by the Sanitary Local Health Unit Rome 3, which is responsible for the health of the sheltered dogs.

Neither anaesthesia nor euthanasia, or any kind of animal suffering, was part of the study. The protocol was carried out in accordance with the relevant Italian guidelines and regulations.

## Results

The inter-observer reliability was measured and it corresponded to r = 0.99 on 5 dogs (9 behavioural patterns).

No dogs showed disease symptoms during the study, except for one dog (Gargamello, under treatment) that had a single episode of diarrhoea, during the second day of T_2_, which disappeared without pharmacological intervention; so we did not exclude this dog from the study. In this study, dogs well tolerated olive oil both with or without the addition of CBD.

The median aggressive levels at T0 looked different for the two groups, but the test for homogeneity applied to the treated and control groups at T0 revealed that this difference was not significant indicating that there was no significant difference in the median level of aggression in the two groups at the start of the study (group A: median = 6.0, IQRs 17–0.75; group B: median = 2.0, IQRs 4.5–0; Mann–Whitney U test, T_0_: Z = 48; N = 24; p = 0.150).

**Figure 1:**
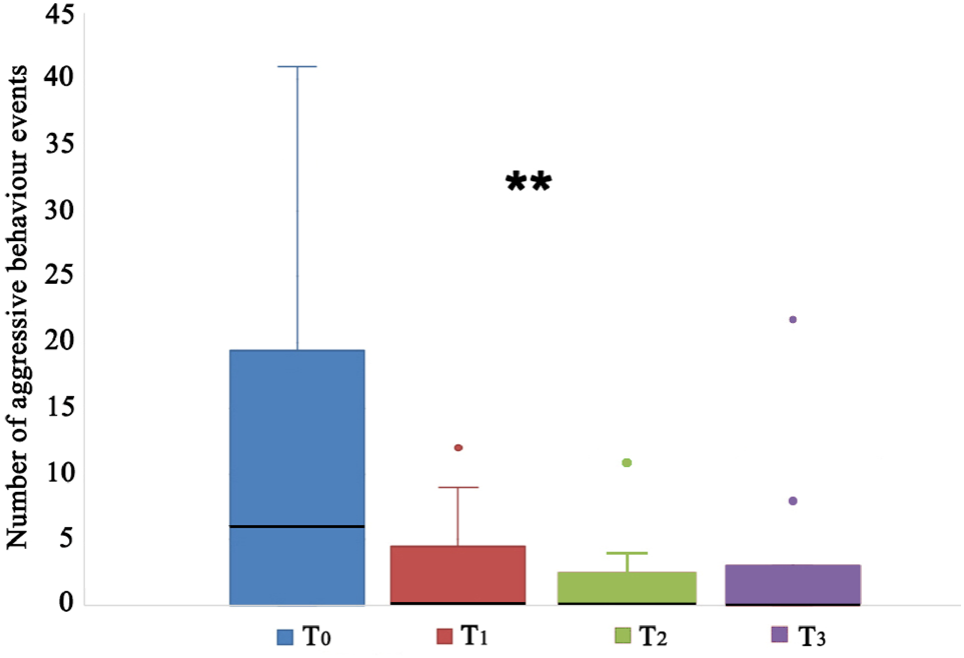
Aggressive behaviour towards humans of dogs treated with cannabidiol (CBD) at the start of the study (T_0_), after 15 (T_1_) and 45 (T_2_) days from the beginning of the treatment, and 15 days after the end of the administration of CBD (T_3_). **p < 0.05; the black bars within the box plots indicate the median; the dots represent the outliers.

Aggressive behaviour towards humans decreased significantly over time in CBD treatment group (Friedman test, T_0_, T_1_, T_2_, T_3_: χ^2^ = 13.300; df = 3; N = 12; p = 0.004). However, in the pairwise comparisons, only the T0-T2 comparison was significant (p = 0.004, adj. sig. p = 0.027) (Fig. 1).

**Figure 2:**
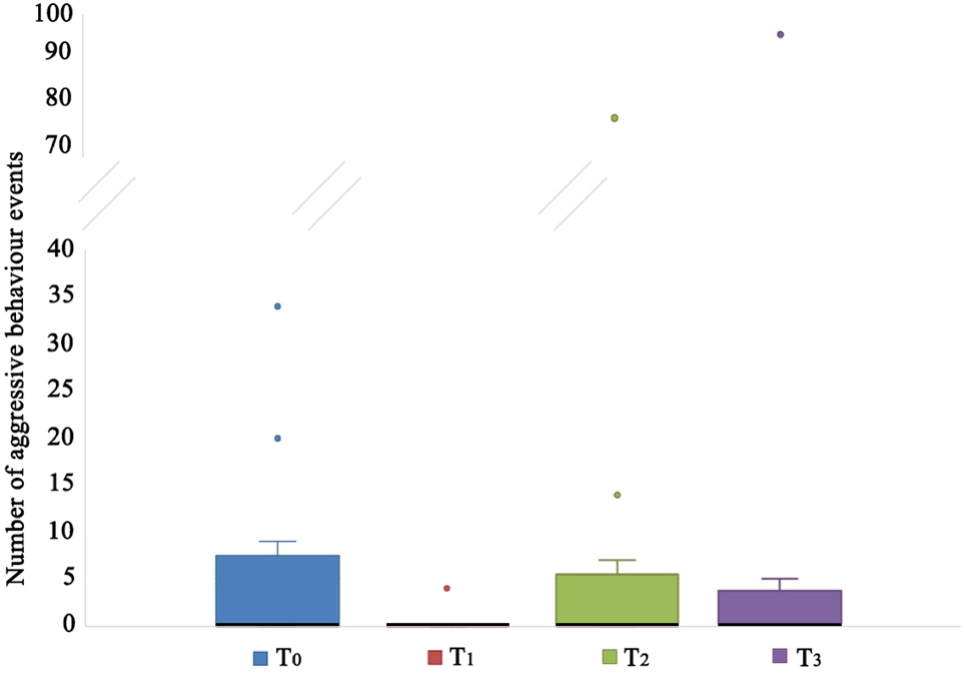
Aggressive behaviour towards humans of dogs receiving olive oil as a placebo at the start of the study (T_0_), after 15 (T_1_) and 45 (T_2_) days from the beginning of the treatment, and 15 days after the end of the administration of olive oil (T_3_). The black bars within the box plots indicate the median; the dots represent the outliers.

On the contrary, in the control group the aggressive behaviour towards humans did not decrease due to the administration of olive oil (without CBD) (Friedman test, T_0_, T_1_, T_2_, T_3_: χ^2^ = 6,268; df = 3; N = 12; p = 0.09; Fig. 2).

**Figure 3:**
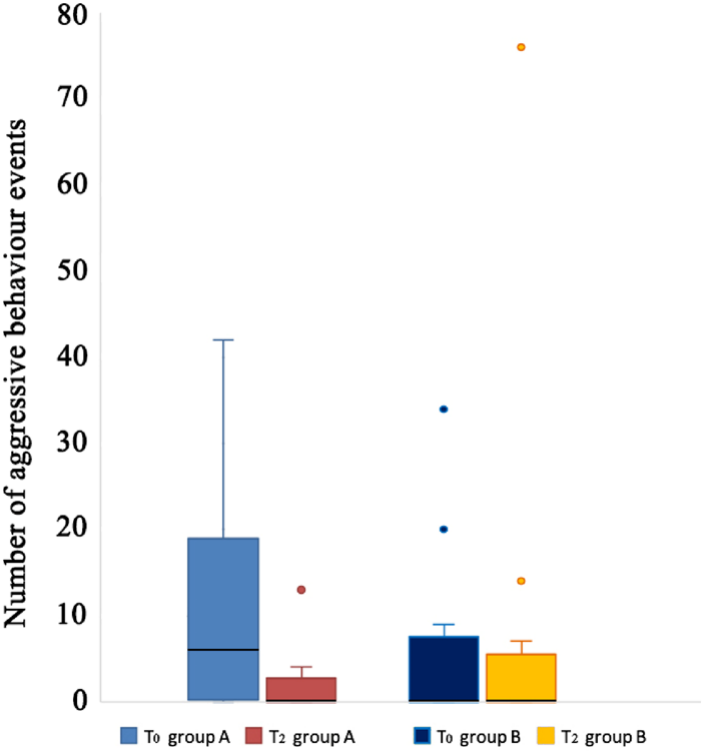
Difference in aggressive behaviour towards humans at different times (T_0_ = before the start of the experiment and T_2_ = 45 days from the start of the experiment) for dogs treated with cannabidiol (CBD) and dogs in the control group (receiving olive oil as a placebo). The black bars within the box plots indicate the median; the dots represent the outliers.

The reduction of aggressive behaviour toward humans was marked in the treated group, but the difference between the treatment and control groups in the decrease of aggressive behaviour towards humans was not significant (Mann–Whitney *U* test, T_2_-T_0_: Z = − 1.81; N = 24; p = 0.078; Fig. 3).

**Figure 4:**
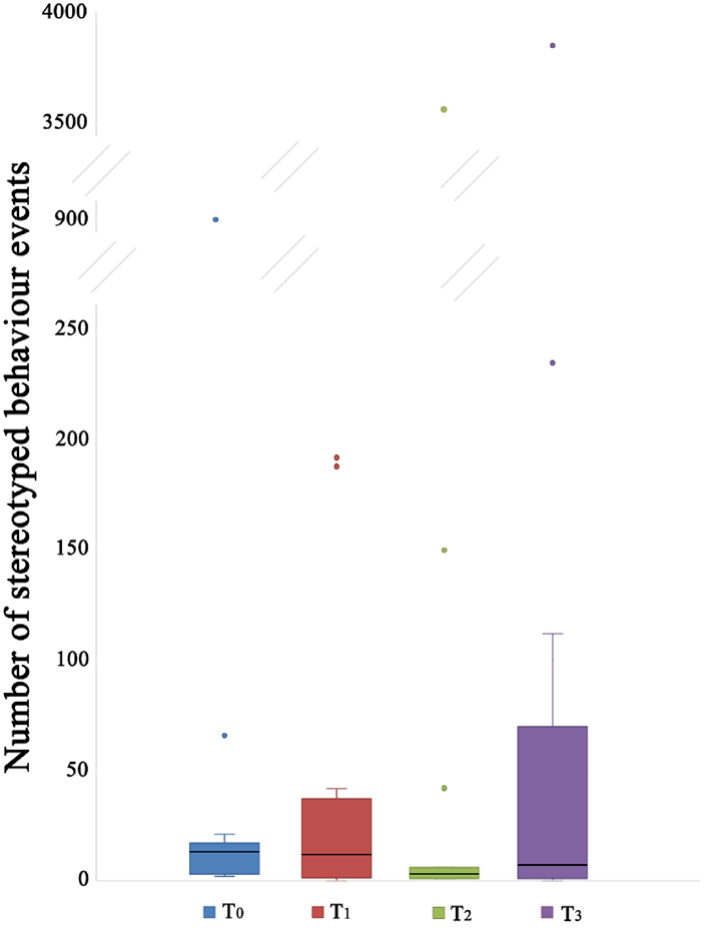
Stereotyped behaviour of dogs treated with cannabidiol (CBD) at the start of the study (T_0_), after 15 (T_1_) and 45 (T_2_) days from the beginning of the treatment, and 15 days after the end of administration of CBD (T_3_). The black bars within the box plots indicate the median; the dots represent the outliers.

**Figure 5:**
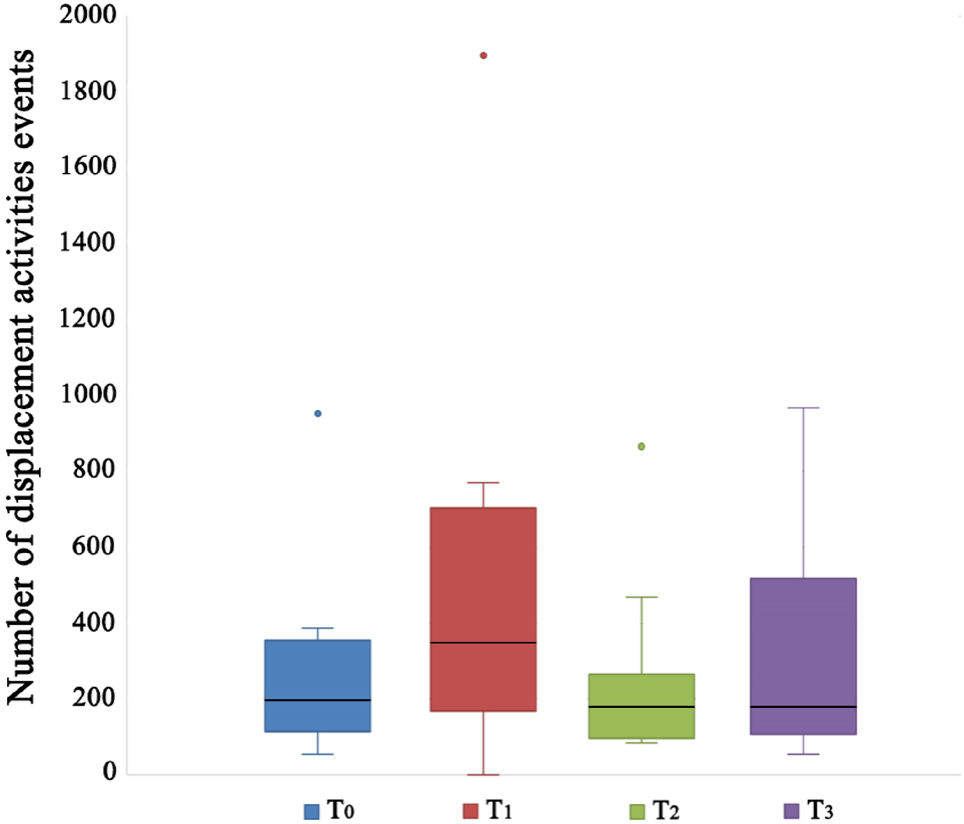
Displacement activities of dogs treated with cannabidiol (CBD) at the start of the study (T_0_), after 15 (T_1_) and 45 (T_2_) days from the beginning of the treatment, and 15 days after the end of administration of CBD (T_3_). The black bars within the box plots indicate the median; the dots represent the outliers.

Concerning the stress related behavioural patterns (stereotyped behaviour and displacing activities), our results did not show any effect of CBD on their frequencies (Friedman test, T_0_, T_1_, T_2_, T_3_: χ^2^ = 2,136; df = 3; N = 12; p = 0.545; Fig. 4; χ^2^ = 0,479; df = 3; N = 12; p = 0.923; Fig. 5).

Finally, the analysis of all behavioural patterns of the dogs, related to attention and interaction with the environment (looking outside/observer/volunteer, raising of ears and looking outside/at observer/at volunteer carefully, dozing, sniffing object/observer/volunteer) suggested that the treatment with CBD did not reduce the level of attention of dogs and did not make them less perceptive of the environment and of the stimuli that surrounded them (Friedman test, T_0_, T_1_, T_2_, T_3_. Attention: χ^2^ = 6,300; df = 3; N = 12; p = 0.09; dozing: χ^2^ = 4,361; df = 3; N = 12; p = 0.225; sniffing: χ^2^ = 3,769; df = 3; N = 12; p = 0.287).

## Discussion

According to the information found in the literature (e.g.,^13,16^), our dogs did not show any of the symptoms referable to CBD intolerance. Daily monitoring of the health of the dogs under observation allowed us to evaluate any eventual pathological responses to olive oil, CBD or both. Given the occasional and rare occurrence of intolerance symptoms (one isolated episode of diarrhea), it is possible to conclude that the olive oil treatment, with or without CBD, was well tolerated.

Although the difference in the decrease of aggressive behaviour between the control and the treated group was not significant, possibly due to the small sample size, our results suggest that the treatment with CBD could reduce the frequency of aggressive behaviour towards humans and highlights the need for further studies.

There are in the shelter, of course, temporal and spatial limitations that vary from shelter to shelter, which could affect the results. As it is well known^43,58^, sheltered dogs in general and the dogs in this study in particular, suffer from inter- and intra-specific social deprivation, total lack of interactions at night, and lack of exercise because they are in cages. In trying to minimize the number of variables that could have affected the results in such a variable environment, we chose dogs that had been in shelter for at least nine months and displayed signs of chronic stress. In fact, dogs entering the shelter have behavioural responses due to acute stress^36,45^. According to these considerations, the results presented here acquire value since they suggest a possibility of response to the treatment in a challenging environment, that could be even greater in an environment where the limitations described above are less present and the possibilities to control the dogs are greater. Many attempts have been made to classify aggressive behaviour in domestic dogs;^59^ combined the descriptive and functional classification system, describing a typical aggression sequence. The same author claims that if the aggression sequence is altered, this indicates that the aggression has reached a pathological level. Additionally, when the frequency of aggressive behaviour is so high that it occurs out of context, becoming unpredictable, it can be considered pathological^50,60^.

The dogs involved in this study were selected because they showed behavioural symptoms that lead to a diagnosis of behavioural disorders and one of the symptoms was excessive aggressive behaviour. Aggressiveness is a very complex phenomenon: the muscles contract, ready for action, the hair stands up, the pupils dilate, the heart beats at a higher rate, blood pressure increases; the rise of the latter carries to all the cells of the body a frantic but surprisingly well coordinated variety of hormones, cytokines and other molecular messengers that inform the cells of the body about the situation: ‘we are going to attack!’.

In general, it would be an erroneous approach to try to ascribe the hyper aggressiveness of a dog to a few causes; moreover, it would be equally wrong and naive to neglect the possibility that the alteration on several levels of the complex system underlying aggression does not cause a chronic state of malaise for the animal. Many of the dogs in this study showed excessively frequent aggressive behaviours. Some of them showed a high level of aggressiveness before entering the kennel, but their permanence in that environment may have increased it or it may have been brought about in dogs that did not present it to start with.

Takahashi et al*.*^61^ suggested that social stress could induce excessive recurrent aggressiveness that becomes maladaptive because it brings about a dysregulation of the immune system. These authors also suggested that the dysregulated immune responses vary according to the rank of the individual, but it was not possible to evaluate this variable in the dogs under study because, due to their high level of aggressiveness, it was necessary to house them individually. What remains beyond doubt is that their behaviour denounced a high level of malaise.

Our results clearly suggest that CBD treatment might be effectively used to improve welfare in dogs housed in a shelter.

However, if CBD treatment causes a reduction in the aggressive behaviour of the dogs, this effect, in turn, might improve the relationships between the dogs and the staff of the kennel, facilitating dog management and increasing the level of dog welfare; in fact, it has been found that walking on a leash or having physical contact with humans improves the level of dog welfare housed in a shelter^58,62,63^.

Other categories widely used to evaluate dogs’ well-being are displacement activities and stereotypies. They are recognized to be a flag of physical and emotional discomfort in humans and in non-human animals^46–49^. Our results did not show any effect of CBD on the reduction of those behavioural patterns. In humans, an antipsychotic activity of CBD was assessed and found to reduce the occurrence of apomorphine-induced stereotypies^64^, but the mechanism by which CBD exerts its anxiolytic effects has not been fully clarified, yet. In rodents, an effect of CBD has been found on stereotyped behaviour because it reduced marble burying behaviour following intraperitoneal administration^19,20^, but this effect was not observed reliably when CBD was administered orally^19^. In this study, the lack of effects on dogs' anxious behaviour attributable to the administration of CBD may be due to oral instead of intraperitoneal administration, as studies on rodents^19,65^ and dogs^66^ have indicated.

In this study, we also did not find any effect of CBD regarding the reduction of displacement activities. However, before discussing this lack of effect, a premise is due. Some authors suggested that displacement activities are behavioural constituents of the adaptive stress response^67^; morphologically, in nonhuman primates these behavioural patterns have something to do with body care: self-grooming, scratching, body shaking, stretching and yawning. They can be associated with different kinds of situations but all situations have in common uncertainty and anxiety as the stressful causal factors^46^; some pharmacological studies, reviewed in^67^, confirmed that displacement activities (mainly scratching) are a valid measure of stress in nonhuman primates and human subjects. In domestic dogs, an indirect suggestion comes from^58^ who found that the frequent display of displacement activities such as self-grooming, scratching and body shaking, are associated with a lower level of antioxidant capacity in shelter dogs. There are very few papers on the effect of different treatments in this behavioural category^68^, for example, did not find an effect of the appeasing pheromone in reducing displacement activities in shelter dogs. Despite the evidence that, through the analysis of some physiological parameters, some drugs reduce the stress level in dogs, such as gabapentin^69^ or clonidine^70^, the drug effect on stress-related behaviour has been neglected. Furthermore, no experiments to investigate the neurobiological correlates of displacement activities and their relationships with negative emotional states have ever been carried out in the domestic dog. Thus, in this species, it is not even clear which behavioural patterns can be considered displacing activities that, in turn, are behavioural components of the adaptive stress response, probably causing anxiolytic effects. Future studies should be focused on both these aspects of neurobiology in domestic dogs.

One of the most robust results of this study is that CBD treatment did not decrease the activity of the dogs studied, as already highlighted for other species^20^. This is an important point because a decrease in dog activity could have reduced aggressive behaviour and biased the results. Dogs under treatment displayed the same level of attention towards the environment before and after the treatment.

Future studies should include a larger sample of sheltered dogs treated with CBD in order to confirm the action of CBD on some behavioural patterns, which would increase the level of dogs’ welfare.

## Conclusions

In this study, we assessed the effects of CBD on dogs’ behaviour. An administration of CBD every 24 h did not result in any effects on behavioural categories related to stress but seemed to reduce aggressive behaviour. Additional investigations are necessary to widen the sample of dogs and to combine a behavioural therapy with CBD administration. Our results pave the way for further behavioural and veterinary studies to understand if CBD could be efficacious also in the treatment of behavioural disorders.

## Supplementary Information

Supplementary Information.
