# Author Correction: *Cannabis sativa* L. may reduce aggressive behaviour towards humans in shelter dogs

**DOI:** 10.1038/s41598-021-03543-x

**Published:** 2021-12-09

**Authors:** Sara Corsetti, Simona Borruso, Livia Malandrucco, Valentina Spallucci, Laura Maragliano, Raffaella Perino, Pietro D’Agostino, Eugenia Natoli

**Affiliations:** 1 School of Agriculture and Environment, The University of Western Australia, Crawley, WA 6009 Australia; 2 Canine Consultant, Rome, Italy; 3 Canile Sovrazonale, ASL Roma 3 (Local Health Unit Rome 3), Rome, Italy; 4 Istituto Zooprofilattico Sperimentale del Lazio e Della Toscana M. Aleandri, Rome, Italy; 5 Canile Pubblico Muratella e Pontemarconi, Roma Capitale (Municipality of Rome), Rome, Italy

Correction to: *Scientific Reports* 10.1038/s41598-021-82439-2, published online 02 February 2021

The original version of this Article contained an error. The company that provided the CBD oil for the study incorrectly described the oil’s characteristics.

As a result, in the Materials and methods section under the subheading ‘Treatment’,

“CBD based oil consisted of an extraction from aerial parts and inflorescences of the plant *Cannabis Sativa* in organic extra virgin olive oil to the proportion of 150 g of CBD in 1 L of oil.”

now reads:

“CBD based oil consisted of an extraction from aerial parts and inflorescences of the plant *Cannabis Sativa* in organic extra virgin olive oil to the proportion of 150 g of *Cannabis sativa* inflorescences and aerial parts in 1 L of oil.”

The original Article has been corrected.

