# Supplementary material for: Cannabis sativa L. may reduce aggressive behaviour towards humans in shelter dogs
Source: Sci Rep. 2021 Feb 2;11:2773. doi: 10.1038/s41598-021-82439-2 (PMC7854708; doi:10.1038/s41598-021-82439-2)
Supplement: Supplementary file 1 — Supplementary Information. [file 41598_2021_82439_MOESM1_ESM.pdf]

Manuscript title: The effects of Cannabis sativa on aggressive behaviour towards humans in shelter dogs.

Corsetti, S.[1*], Borruso, S. [2], Malandrucco, L.[3], Spallucci, V.[4], Maragliano, L.[3], Perino, R. [3], D'Agostino, P. [5] & Natoli, E[3].

[1]School of Agriculture and Environment, University of Western Australia, 6060 Crawley, WA.
[2]Canine consultant, freelance, Rome, Italy.
[3]Sovrazonal Dog Shelter, Local Health Unit Rome 3, Rome, Italy.
[4]Istituto Zooprofilattico Sperimentale del Lazio e della Toscana *M. Aleandri*, Rome, Italy.
[5]Public Dog Shelter Muratella e Pontemarconi, Roma Capitale, Rome, Italy

**Corresponding author:* sar.corsetti@gmail.com

Supplementary Information

Ethogram utilised during the sessions of behavioural observation (Corsetti et al. 2018).

**Activity:**
Standing: staying in an upright position, on four legs.
Walking: walking in the fenced area within the shelter.
Trotting: trotting in the fenced area within the shelter.
Galloping: galloping in the fenced area within the shelter.
In/out: going in and out of the indoor/outdoor zone of the cage.
**Aggressive behaviour:**
Growling: threatening vocalisation coming from the throat.
Sideways glance: looking transversely with the head upright or bent. The glance is threatening.
Raising fur: raising the fur of the head, body and tail so that the dog appears to have a larger size and is thus more threatening.
Curling lip: light raising of the upper lip, usually only on one side, with a threatening partial display of the teeth.
Showing teeth: curling of the upper and lower lips while opening the mouth with a threatening display of the teeth, particularly the canine teeth.
Dashing at bars: dashing at bars in the direction of the observer, of another person or of another dog.
**Displacing activities:**
Body shaking: shaking the body quickly sideward.
Scratching: raising one hind leg and vigorously scratching part of the body.
Muzzle licking: passing the tongue over the muzzle.
Auto-grooming: cleaning itself with the tongue and the teeth.
**Stereotyped or repetitive behaviour:**
Repetitive pacing in circles: repetitive walking in a circle within the cage.
Licking or biting compulsively: repeatedly licking or biting the bars, the wall and objects.
Catching flies: trying to catch an imaginary fly with the mouth, clutching at empty air with the teeth.
Coprophagy.
Self-mutilation: licking itself continuously in same part of the body, so intensely to cause abrasions or even wounds.

**Attention:**
Raising ears
Looking outside: looking outside the cage.
Looking out carefully: looking outside the cage very carefully; the position resembles that described for "prompt" but the dog is not ready to spring up.
Looking at observer: looking at the observer.
Looking at unknown people: looking at people the dog does not know.
Looking at volunteer: looking at a shelter volunteer worker.
Looking at dog: looking at another dog.
Raising foreleg: raising one foreleg.
Raising forelegs on wall: raising both forelegs onto the wall or onto the bars, looking carefully outside
**Olfactory investigation:**
Sniffing environment: putting the muzzle on the ground, on the wall, or on the objects in the cage, the dog sniffs the environment.
Sniffing air: raising the head, moving the nostrils and breathing the air to perceive odours.
Sniffing observer: pointing the muzzle towards the observer, the dog moves the nostrils clearly trying to perceive the odours of the observer.
Sniffing unknown people: pointing the muzzle towards people the dog does not know, the dog moves the nostrils clearly trying to perceive their odours.
Sniffing volunteer: pointing the muzzle towards volunteers working in the shelter, the dog moves the nostrils clearly trying to perceive their odours.
Sniffing dog: pointing the muzzle towards another dog, the subject moves the nostrils clearly trying to perceive the object's odours.
**Dominant behaviour:**
Staring: gazing at the observer, another person or another dog right in the eyes.
Stiff body and tail still: standing still in an upright posture, with the ears raised and turned forward, the four legs straight and rigid and the tail immobile and rigid at a medium height.
Raised tail: the tail is held high while it is still.
Wagging with the tail held high: moving the tail sideward while held high.
Pricked-up ears: holding the ears forwards while assuming an upright body posture with head and tail held high, legs straight and stiff.
Paw or a muzzle on a conspecific's back: putting the muzzle or one forepaw or both over the back of another dog.
**Submissive behaviour:**
Avoiding eye contact: looking away from the observer, another human or another dog, who is looking at the subject.
Lowering head: lowering the head in front of the observer, another human or another dog.
Ears down: putting the ears down, pressed on the head, or holding them backwards.
Cringing: lying with the ventral region in contact with the ground.
Tail between the legs: holding the tail down or tightly between the hind legs and against the belly.
Lying down on back: laying down on the back exposing the ventral side of the chest and sometimes the abdomen.
**Vocal communicability:**
Barking: emitting an abrupt, loud, noisy, and often repetitive vocalisation characteristic of dogs.<br>Mumbling: emitting a vocalisation that consists of a sort of inside murmur.
Whining: emitting a mournful vocalisation.
Grumbling: emitting a low and deep vocalisation that seems to come from the chest, the dog generally has the mouth closed.

Howling: emitting a vocalisation that consists of a long, high and mournful sound; characteristic of wolves, quite rare in dogs.
Snorting: emitting a vocalisation while puffing out its cheek and emitting air.
**Affiliative behaviour:**
Wagging tail: the tail is wagged sideward but not held high, in a relaxed manner.
Giving the foreleg: raising one of the forelegs and leaning it in the direction of the observer.
Leaning on bars: leaning the body in direct contact with the bars of the cage.
Licking the mouth: licking the muzzle of other dogs.
Passive contact: staying in contact with some part of the body, without sleeping.
Allo-grooming: cleaning the fur of another dog, licking and nibbling.
**Resting:**
Sitting: sitting down with the rump leaning on the ground.
Lying: lying down on the ground.
Dozing: curling up, the dog is half asleep.
**Playing:**
Inviting to play: inviting another dog or human to play: the dog bends down with the forelegs outstretched on the ground and the rump upwards, or brings an object, runs around and jumps.
Answering invitation to play: playing with another dog after having been invited to do so.
